# To determine the level of satisfaction among medical students of a public sector medical university regarding their academic activities

**DOI:** 10.1186/1756-0500-4-380

**Published:** 2011-10-05

**Authors:** Bushra Manzar, Nabeel Manzar

**Affiliations:** 1 Dow University of Health Sciences, Karachi, Pakistan

**Keywords:** Students, Satisfaction, Dissatisfaction, Medical Education, Curriculum, Academic Activities

## Abstract

**Background:**

An ongoing evaluation system is essential to determine if the academic system in place has worked to produce a better product, hence the objective of our study was to evaluate the satisfaction level among medical students regarding their academic teaching and assessment method and what measures will they suggest for the future to rectify the current situation.

This questionnaire based cross sectional study was conducted in a public sector medical university from February to July 2010. A well structured questionnaire was administered to a random sample of 375 final year medical students. However 292 of the students provided informed consent and filled in the questionnaire which included their demographic profile as well as questions in line with the study objective. Data was entered in a Statistical Package for Social Sciences (SPSS version.16) and analyzed using descriptive statistics.

**Findings:**

The male to female ratio in our study was 1:2. Most of the students (57.2%) were dissatisfied with the quality of teaching in the university. Fifty-seven percent of the participants believed that the current standard of their institute were not at par with those of international medical universities. BCQ's were the mode of examination questions preferred by the majority of the students. Most of the students (66.1%) wanted the university to conduct career planning seminars to help them plan their career.

**Conclusions:**

These results suggest that the students of public sector medical universities are unsatisfied from current academic facilities and teaching activities. Students recommend increased emphasis on better lectures and practical training as well as a need to incorporate career planning sessions for the students to help plan them their future career paths.

## Findings

The male to female ratio in our study was 1:2. Most of the students (57.2%) were dissatisfied with the quality of teaching in the university. Fifty-seven percent of the participants believed that the current standard of their institute were not at par with those of international medical universities. BCQ's were the mode of examination questions preferred by the majority of the students. Most of the students (66.1%) wanted the university to conduct career planning seminars to help them plan their career.

## Introduction

The medical training in Asia is traditionally teacher centric and relies on hospital based training [1]. Pakistan is a developing third world country with an average educational system and is no different in this aspect. It devotes a nominal amount of its budget to education due to limited amount of resources and the global recession. Despite these disadvantages a number of steps have been taken to modify the curricula especially of medicine consistent with that of international standards by the individual universities. These modifications include introduction of better system of evaluation and teaching.

An ongoing evaluation system is essential to determine if the new system is working to produce a better product [2]. The relationship between the administrative structures of the medical school and the teaching hospitals, the other responsibilities of teachers and administrators, and the intricacies of the curriculum as a system of inter related components means that any new change has wide repercussions [3]. The students are the direct beneficiary or benefactor of the system and today in many parts of the world graduates are required to complete an assessment of their curricular program for evaluation and feedback [4,5]. In the United States, Graduate Exit Questionnaire (GEQ) is a part of the routine educational process. Medical graduates' evaluation of the educational program in GEQ is utilized for quality assurance and curriculum revision. Curriculum evaluation includes gathering information about the merits and demerits of the educational program. This monitoring helps to determine whether corrective measures are indicated [6].

There is a scarcity of data about satisfaction level among medical students in regards to their academic activities. This is associated with impaired learning in third world countries like Pakistan, where resources are limited and budgetary allocation to health inadequate. In this scenario our study carries huge implications for revolutionizing teaching and assessment patterns in medical training. This could potentially be due to the fact that very few studies to evaluate the satisfaction level have been carried out in the context of the local student population in underdeveloped countries where the prevalence of satisfaction levels is not known due to secondary importance being attached to education. Hence to determine the discrepancies and shortfalls in the current academic system of the largest public sector medical university in Pakistan namely Dow University of Health Sciences, a comprehensive study was carried out to establish the student level of satisfaction with the curriculum and what measures can be placed in the future to rectify the situation.

## Methods

This questionnaire based, cross sectional study was carried out at the constituent colleges of Dow University of Health Sciences (DUHS), Karachi, Pakistan namely; Sindh Medical College (SMC), Dow Medical College (DMC) and Dow International Medical College (DIMC) for period of 6 months from February to July 2010. A total of 292 final year medical students undergoing training at the university were enrolled in the study through random sampling according to the set out inclusion criteria. The study protocol was reviewed and approved by an ethics committee at the study centre and the study was carried out in accordance with the declaration of Helsinki of 1975 as revised in 1983. The primary outcome measure was to determine the level of satisfaction from medical students regarding the current curriculum, the academic system and what future changes they envisage in order to improve the educational program.

The participants fulfilling the following set out criteria were enrolled in the study:

1. Informed consent from the university students for participating in the study

2. Participants of all ages and gender as determined by filled in proformas

3. Supervised questionnaire administration by the co-investigators

4. Completely filled proformas regarding socio-demographic information

First the study objectives were explained to the participants. Informed consent was taken and full confidentiality was assured to the participants. They were made to fill out a pretested questionnaire which comprised of close ended questions in line with the study objective to assess student's opinions and expectations concerning DUHS academic curriculum. The questions on the questionnaire were carefully derived from other similar questionnaires at the international level like the graduate exit questionnaire in line with the study objective. The five year academic system of DUHS in medical colleges leads to a degree called Bachelor of Medicine and Bachelor of surgery (MBBS). Each academic year is further split into two semesters. The first three years focus on basic medical training followed by two years of hospital based practical training. The curriculum is based on international books prescribed for the purpose of information and knowledge. The assessment system used is based on MCQ/BCQ theoretical paper followed by Object Structured Clinical/Practical Examination (OSCE/OSPE). The same undergraduate system of medical education is being utilized in other Asian countries like India and others and is widely recognized in developed countries for postgraduate studies and practice purposes. The study questionnaire was pretested. The students satisfaction rate was measured by a 5 point Likert type scale i.e., a- strongly agree, b- agree, c- disagree, d- strongly disagree, e-no idea. The non-medical, nursing and students of dental sciences were excluded from the study. The questionnaires were administered under supervision of the co-investigators and the participants were fully explained the meaning of the questions prior to filling in the questionnaires so that everybody understood the same meaning and hence bias in answers attributed to different understanding of questions was diminished to a large extent.

Classification of the subjects was done according to gender and medical college in which they were currently enrolled criteria (Table 1). Data were entered in a Statistical Package for Social Sciences (SPSS version 16) and analysis of the data was done using frequencies, proportions, group means and standard deviations. Descriptive analysis was used to assess the different variables in the study.

**Table 1:** Baseline characteristics of the participants

Variable	No. of Subjects	Frequency (%)
**Mean Age (year ± std.dev)**	24.2 ± 1.3	

**Gender**		
Male	101	34.6
Female	191	65.4

**Constituent College**		
DMC	123	42.1
SMC	123	42.1
DIMC	46	15.8

## Results

A total of 375 final year medical students based on random sampling were accessed to participate in the study. However, only 292 out of 375 students consented to be included in the study based on the inclusion criteria. The response rate was 77.86%. Out of 292 respondents, there were 101 males (34.6%) and 191 females (65.4%) aged between 20 and 25 years (mean age = 24.2 years). The male to female ratio in our study was 1:2 reflecting the gender composition at the participating colleges. Table 1 shows the baseline characteristics of the participants.

The central idea of the current research was whether the students are generally satisfied with the curricular activities in their respective colleges and hence a graded system was used to assess the responses of the respondents ranging from strongly agree to having no idea on the particular question. There was no significant difference among the medical students of different colleges with respect to their views on satisfaction in regards to their curricular activities and hence we have presented the results in totality rather than further stratification. Satisfaction rate for the sake of simplicity has also been presented as a combination of strongly agree, agree or strongly disagree, disagree.

In our study majority of the participants (57.5%) believed that the current standard of their institute were not at par with those of international medical colleges. Similarly most of the students were dissatisfied with the quality of teaching (57.2%) and also with the pattern of typical lecture based teaching (63.7%) at their respective colleges. Most of the students (77.1%) thought multimedia to be the most effective teaching tool followed by transparencies and traditional blackboard teaching. Majority of the students (72.9%) believed that 30 minutes was the ideal duration time for a lecture. Similarly most of the participants (64%) wanted the preclinical curriculum to be revamped as it was not clinically oriented. One hundred and seventy eight respondents (60.9%) believed that the preclinical teaching was inadequate as compared to their clinical teaching.

Greater than 50% of the students believed that the new semester system as well as the current method of conducting the Object Structured Practical Examination (OSPE) and Object Structured Clinical Examination (OSCE) was a fair mean of assessment and not only helped eliminate the bias prevalent in the old viva system but also helped in improving the clinical skills by regular assessment. One hundred and forty-six students (50%) favored the best choice question (BCQ) system of assessment. Students view regarding the curricular system is shown in table 2.

**Table 2:** Students view regarding curricular system

Question	Strongly agree	Agree	Disagree	Strongly Disagree	No idea
**Standard of your institute matches with the international standards of medical college**	6.8	30.1	24.3	33.2	5.5

**The quality of teaching and lectures in the university is satisfactory**	5.8	33.2	17	40.2	3.8

**Current teaching practices i.e. lectures help ace local examination as well as international exams like USMLE & PLAB etc**	5.1	20.9	25.8	37.9	10.3

**You are satisfied with the semester system**	00	82.5	9.8	4.6	3.1

**Multiple mode's of assessment help improve your knowledge and clinical skills**	16.8	55.8	11.1	7.4	8.9

**The pattern of OSCE/OSPE is satisfactory**	00	59.6	21	9.1	10.3

**Best form of assessment is**	**MCQ **36	**BCQ **50	**Short Q/A **4	**Descriptive **8	**No Idea **2

USMLE: US medical licensing examination; PLAB: Professional and Linguistic Assessments Board; OSPE: Object Structured Practical Examination; OSCE: Object Structured Clinical Examination; MCQ: Multiple Choice Question; BCQ: Best Choice Question.

Ninety-two percent of the students wanted the college administration to build a lending library and a bone bank to facilitate the students. Similarly 42.5% of the students were discontented with the old computer systems in the digital library and wanted them to be updated with new software. Most of the students also wanted the university to conduct career planning seminars to help them plan their career. Most of the students (75.5%) believed that they will go on to become successful doctors in the future while 79.1% of the students wanted to serve their home institution in the coming years for its betterment. Student's suggestions for improvement in the current system and their views about their future are shown in Table 3.

**Table 3:** Students suggestions for improvement in regards to academic activities

Question	Strongly agree	Agree	Disagree	Strongly Disagree	No idea
**University should have a lending library/bone bank**	51.4	41.4	6.8	00	0.3

**Up to date computers should be provided in your digital library **	10.6	41.8	10.2	32.3	5.1

**Career counseling help should be provided by the university**	5.5	24.7	11.4	54.7	3.8

**Future wellbeing of your university is also a priority **	34.1	45	20.9	00	00

## Discussion

This study highlights the major factors that adversely affect the curricular activities and satisfaction rate among medical students in a public sector medical university of a developing country like Pakistan. Recommendations were also sought from the students as they provide an invaluable source for improving current medical education based on learner's opinion [7,8]. Seventy Eight percent of the respondents based on the inclusion criteria were incorporated in the study. Eighty three students did not consent to participate in the study because majority of them were of the opinion that their responses would not be able to change the current system. This was in contrast to the opinion held by the majority of the study participants. There were a majority of female respondents in the study which is in line with the fact that today more and more females and fewer males are adopting medicine as a profession. This may be due to the difference in remunerations being offered in medicine as compared to other high yield fields of study.

Forty-three percent of the respondents were satisfied with their medical education. A satisfaction rate of 28.4% and 90.2% for Iran and USA respectively have been noted on GEQ [9,10]. Most of the students were not satisfied with the current level of teaching patterns in the university which include the traditional lecture based learning. Respondents were of the view that Problem Based Learning (PBL) and small group discussions could be more helpful than didactic style of teaching [11-13]. In a recent study carried out by Nandi et al, they found that students of the newer problem-based learning curriculum found learning to be "more stimulating and more humane" whereas students of the conventional curriculum found learning to be "nonrelevant, passive, and boring" [14].

Similar results have been documented in studies carried out by Fischer et al and Costa et al where they found that interactive style of teaching and group discussions were more preferred by the undergraduate students than lecture based didactic learning [15,16]. Majority of the students favored multimedia as a supporting teaching tool as compared to old blackboard teaching and transparencies. Similar results have been expressed in numerous studies through-out the globe [17].

Many students found 30 minutes to be adequate for a lecture and were of the opinion that any teaching beyond the 30 minutes led to over saturation and excess information. Increased lecture time has also been cited in many studies for skipping lectures [17]. In a survey conducted by Trevena, it was noted that students felt that self directed learning in basic and clinical sciences was more effective than lectures [18]. Participants were of the view that the curriculum taught in medical colleges today especially of basic sciences like anatomy and biochemistry was outdated and was without clinical correlate and also did not help them in acing international examinations. Most of the respondents (85%) were knowledgeable about the USMLE and other international examination and were of the opinion that current method of teaching will not be able to help them in those examinations. Although none of the students had given the USMLE they still felt that pattern of questions on international exams were more clinically relevant as compared to non-clinical curriculum taught to them in medical school. This concern was also raised by the students in a Graduate Exit Questionnaire (GEQ) survey, where according to them lack of integration and coordination in basic science courses, dissociation and lack of relevance of the curriculum were major drawbacks to the medical programme [9]. Similarly medical students have also complained of being significantly more dissatisfied with their basic science teaching than with their clinical teaching and training. In a study done at the University of Saskatchewan, Canada, a majority of senior undergraduate students indicated that they did not remember much from their first year courses and the preclinical teaching content was not relevant to later clinical work or studies, with statistically significant effect of knowledge loss [19].

Respondents were happy with the new changes that were introduced by the university like the semester system and the multiple modes of assessment including BCQ method of testing and clinical skills evaluation examination on the patterns of OSCE and OSPE. Similar findings have been revealed by Abraham et al, where students suggested a need for multiple modes of assessment rather that single mode of assessment which in their view could not fulfill assessment of all aspects of student's knowledge effectively [20]. In the view of the students introduction of the semester system enabled the students to focus better on their studies due to pressure of regular performance in exams. Students were also of the view that OSCE was the standard method of evaluating clinical skills today in a neutral environment as compared to the old viva voce pattern of examination which in their view was biased. Most of the students favored BCQ method of assessment as in their view it promoted critical thinking. In a study carried out by Oyebola et al, students preference were slightly different with the majority of the participants in the study supporting MCQ based assessment [21].

In this study, we asked respondents what they considered to be the most important factors to be implemented in improving our medical education. Most of the respondents were of the opinion that better qualified teachers, integration of basic science with clinical cases and hands on training were indispensable to a better medical education program. Similar findings have been noted by different studies worldwide [18,22,23]. Majority of the participants were of the view that curricular support facilities like lending library, digital library and bone bank for studying anatomy specimens were inadequate in the university. Most of the respondents being dependant on their parents as well as due to their poor socio-economic profile were not in a position to buy expensive international books and wanted the university administration to build these resources for them. Most of the participants were computer literate and thought that a modular course on information technology should be introduced by the university to help those students not familiar with computers. They were also of the view that computer were indispensable part of health care in the modern era and should be upgraded with softwares to help them conduct research and increase their knowledge in regards to modern day to day advances in health sciences. Access to the internet and internet sources of health information is rapidly increasing in medical universities of Asian origin [24]. The respondents were also dissatisfied with the university for not conducting career planning and future guidance seminars for the students. Many students were confused in regards to their choice of future field of study and were of the view that their uncertainty could be resolved with proper counseling. However counseling and career planning is a rare phenomenon in developing countries of Asian origin where no such facility exists even at the university level. Despite the confusion regarding their future field of study most of the respondents were confident of their success in the years to come while others were more skeptical. Most of the students held this view because of the importance of self study and self belief in their minds. It has to be kept in mind that the education system in Pakistan as other Asian countries is not upto the level of those present in developed countries. Still Pakistan produces one of the best doctors in the world practicing in developed countries like UK. Students believe that even if the system is not that good and they are not satisfied with it, they can still do better with their own hard work. A minority of females knew that their future fate would be to serve as housewives due to early marriage. Another phenomenon so common in eastern countries. Participants were prepared to serve their alma-mater in the future for its betterment.

In conclusion this study fulfills the objective set by the study protocol for this project of assessing the satisfaction and concerns among medical students in regards to curricular activities carried out in the largest public sector medical university in Pakistan. The study presents results from a public sector medical university and therefore limits generalization for private medical colleges where situation may be different as well as the questionnaire design in regards to certain questions is a limitation where future studies can improve upon it. Nonetheless this study holds important implications for policy makers for the future and will help them determine what future strategies and planning need to be undertaken to tackle student's problem and contentment with their curriculum at the grass root level. Updated revisions should be incorporated in the medical curriculum to enable the future medical personals to play a viable role in the future research and development.

## Competing interests

The authors declare that they have no competing interests.

## Authors' contributions

NM and BM conceived the study, participated in its design and coordination. BM performed the data collection and statistical analysis. NM and BM drafted the manuscript. All the authors read and approved the final manuscript.
